# Adult onset asthma and interaction between genes and active tobacco smoking: The GABRIEL consortium

**DOI:** 10.1371/journal.pone.0172716

**Published:** 2017-03-02

**Authors:** J. M. Vonk, S. Scholtens, D. S. Postma, M. F. Moffatt, D. Jarvis, A. Ramasamy, M. Wjst, E. R. Omenaas, E. Bouzigon, F. Demenais, R. Nadif, V. Siroux, A. V. Polonikov, M. Solodilova, V. P. Ivanov, I. Curjuric, M. Imboden, A. Kumar, N. Probst-Hensch, L. M. Ogorodova, V. P. Puzyrev, E. Yu Bragina, M. B. Freidin, I. M. Nolte, A. M. Farrall, W. O. C. M. Cookson, D. P. Strachan, G. H. Koppelman, H. M. Boezen

**Affiliations:** 1 University of Groningen, University Medical Center Groningen, Department of Epidemiology, Groningen, the Netherlands; 2 University of Groningen, University Medical Center Groningen, Groningen Research Institute for Asthma and COPD (GRIAC), Groningen, the Netherlands; 3 University of Groningen, University Medical Center Groningen, Department of Pulmonology, Groningen, the Netherlands; 4 Division of Respiratory Sciences, Imperial College, London, United Kingdom; 5 Population Health and Occupational Disease, Imperial College, London, United Kingdom; 6 MRC-PHE Centre for Environment and Health, Imperial College, London, United Kingdom; 7 Institute of Medical Statistics and Epidemiology (IMSE), Klinikum Rechts der Isar, Technical University, Munich, Germany; 8 Comprehensive Pneumology Center (CPC), Institute of Lung Biology and Disease (iLBD), Helmholtz Center Munich, Neuherberg, Germany; 9 Centre for Clinical Research, Haukeland University Hospital, Bergen, Norway; 10 Univ Paris Diderot, Sorbonne Paris Cité, Institut Universitaire d’Hématologie, Paris, France; 11 INSERM, UMR-946, Paris, France; 12 INSERM, U1168, VIMA: Aging and chronic diseases, Epidemiological and public health approaches, Villejuif, France; 13 Univ Versailles St-Quentin-en-Yvelines, UMR-S 1168, Montigny le Bretonneux, France; 14 INSERM, IAB, Team of Environmental Epidemiology applied to Reproduction and Respiratory Health, Grenoble, France; 15 Univ. Grenoble Alpes, IAB, Team of Environmental Epidemiology applied to Reproduction and Respiratory Health, Grenoble, France; 16 CHU de Grenoble, IAB, Team of Environmental Epidemiology applied to Reproduction and Respiratory Health, Grenoble, France; 17 Kursk State Medical University, Department of Biology, Medical Genetics and Ecology, Kursk, Russian Federation; 18 Department of Epidemiology and Public Health, Swiss Tropical and Public Health Institute, Basel, Switzerland; 19 University of Basel, Basel, Switzerland; 20 Wellcome Trust Centre for Human Genetics, University of Oxford, Oxford, United Kingdom; 21 Siberian State Medical University, Tomsk, Russia; 22 Research Institute of Medical Genetics, Tomsk NRMC, Russia; 23 Population Health Research Institute, St George's, University of London, London, United Kingdom; 24 University of Groningen, University Medical Center Groningen, Department of Pediatric Pulmonology and Pediatric Allergology, Beatrix Children’s Hospital, Groningen, the Netherlands; Kunming Institute of Zoology, Chinese Academy of Sciences, CHINA

## Abstract

**Background:**

Genome-wide association studies have identified novel genetic associations for asthma, but without taking into account the role of active tobacco smoking. This study aimed to identify novel genes that interact with ever active tobacco smoking in adult onset asthma.

**Methods:**

We performed a genome-wide interaction analysis in six studies participating in the GABRIEL consortium following two meta-analyses approaches based on 1) the overall interaction effect and 2) the genetic effect in subjects with and without smoking exposure. We performed a discovery meta-analysis including 4,057 subjects of European descent and replicated our findings in an independent cohort (LifeLines Cohort Study), including 12,475 subjects.

**Results:**

First approach: 50 SNPs were selected based on an overall interaction effect at p<10^−4^. The most pronounced interaction effect was observed for rs9969775 on chromosome 9 (discovery meta-analysis: OR_int_ = 0.50, p = 7.63*10^−5^, replication: OR_int_ = 0.65, p = 0.02). Second approach: 35 SNPs were selected based on the overall genetic effect in exposed subjects (p <10^−4^). The most pronounced genetic effect was observed for rs5011804 on chromosome 12 (discovery meta-analysis OR_int_ = 1.50, p = 1.21*10^−4;^ replication: OR_int_ = 1.40, p = 0.03).

**Conclusions:**

Using two genome-wide interaction approaches, we identified novel polymorphisms in non-annotated intergenic regions on chromosomes 9 and 12, that showed suggestive evidence for interaction with active tobacco smoking in the onset of adult asthma.

## Introduction

Exposure to environmental tobacco smoke increases the risk to develop asthma in childhood [1]. However, the role of active tobacco smoking in the onset of adult asthma remains inconclusive. Current and former smokers have a lower lung function [2–4] and increased bronchial hyperresponsiveness [5], whereas active smoking increases asthma severity [6]. The evidence for new onset asthma after active tobacco smoking is less clear. Active tobacco smoking has been associated with the onset of adult asthma [7,8], but not in all studies [6,9,10]. It has been hypothesized that tobacco smoking moderates the immune system by increasing IgE levels, thereby contributing to asthma onset [11].

Asthma is a complex disease that is thought to be caused by an interaction of environmental exposures and genetic susceptibility. Active tobacco smoking may increase the risk for asthma in a susceptible population only. Two candidate gene studies have suggested an interaction between active tobacco smoking and genetic variants in the occurrence of asthma in adults, i.e. the genes thymic stromal lymphopoietin (*TSLP*) [12] and filaggrin *(FLG)* [13]. Similarly, a study showed an interaction between active tobacco smoking and genes involved in lung function decline [14]. Above studies were based on hypothesis driven gene selection. One genome-wide association study on adult onset asthma, with a hypothesis free design, revealed that polymorphisms in the *HLA-DQ* gene increase the risk for adult onset asthma [15], an effect that was independent of tobacco smoke exposure.

Insight in the interaction between active tobacco smoking and genetic susceptibility is crucial for further development on knowledge on the etiology of adult onset asthma and for the development of effective strategies for asthma prevention. We therefore performed a genome-wide interaction (GWI) analysis using data of studies participating in the GABRIEL consortium [15] We replicated our top hits in a large population study in the Northern part of the Netherlands: LifeLines Cohort Study [16]. We set out to identify new genetic variants that interact with active tobacco smoking with respect to asthma onset at adult age.

## Methods

### Subjects

**Fig 1:**
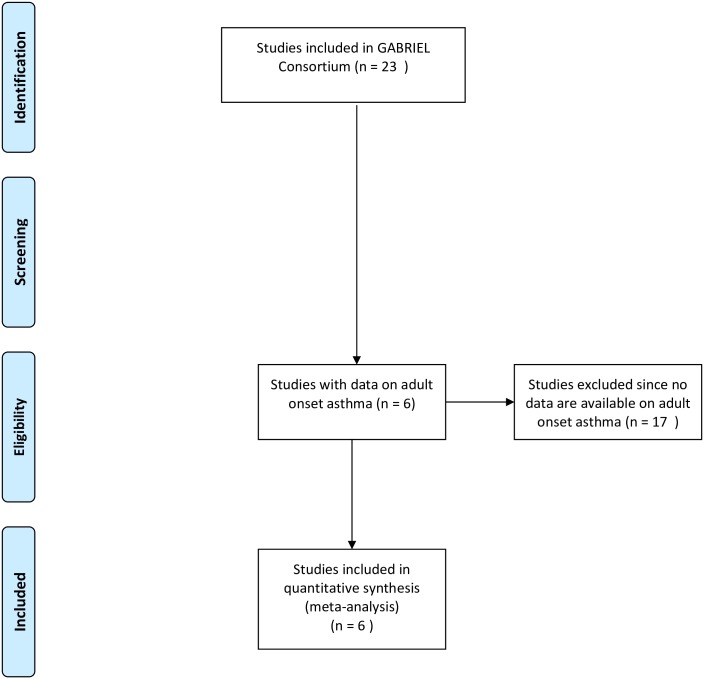
PRISMA flow diagram.

Data from six individual studies selected on presence of adult onset asthma data were included in the discovery meta-analysis on the interaction between single nucleotide polymorphisms (SNPs) and ever active tobacco smoking (Fig 1, S1 and S2 Checklists). All cases and controls were of European descent and two studies had a family structure. The study was approved by the local Medical Ethical Review Committees and all subjects gave written informed consent (Description of studies and ethical approval in the supporting information (S1 File)). Adult onset asthma was defined as asthma diagnosed by a doctor when the subject was 16 years of age or older, as defined within the GABRIEL consortium [15]. Controls were all free of asthma, including childhood onset asthma. Active tobacco smoking was defined as ‘ever active tobacco smoking’. Details on the outcome and exposure definition for the individual studies can be found in the S1 File.

### Genotyping and quality control

Genotyping was performed using the Illumina Human610 quad array (www.illumina.com) at CEA-Centre National de Génotypage, Evry, France. Details on the genotyping method have been described previously [15]. We restricted our meta-analyses to SNPs fulfilling the following quality control criteria in each study: genotype missing rate <3% in cases and controls, minor allele frequency >5% in controls and consistency with Hardy-Weinberg equilibrium in controls (p-value>10^−4^). Samples with >95% genotyping success rate were included in the analyses. We excluded putative non-European samples, identified using EIGENSTRAT2.0 software.

### Statistical analyses

All individual studies were analysed using a logistic regression model with adult onset asthma as outcome. For each individual study a genome wide analysis on adult onset asthma was performed using logistic regression analysis including the SNP, ever active tobacco smoking, as well as the interaction between the SNP and ever active tobacco smoking to assess whether the effect of smoking on adult asthma differed between subjects with different genotypes. Also a stratified analysis was performed to analyse the genetic effect in exposed and non-exposed subjects. In all models an additive genetic model was used. Gender, age and informative principal components for within-Europe diversity were included as covariates. For the studies containing family data, a cluster variable indicating the family relations was included.

We meta-analysed the results of the individual studies (discovery meta-analysis) and used two selection procedures to identify SNPs that interact with ever active tobacco smoking in the adult onset asthma. To assess heterogeneity Cochran’s Q statistic was calculated of each SNP and a random effect model was fitted.

Firstly, we followed the classical GWI study approach that is based on selection of the most significant interaction effect, i.e. the overall difference between the genetic effect in smokers and non-smokers with the lowest p-value. With this approach, smaller genetic effects occurring only after exposure to active tobacco smoking can be missed. For that reason we also followed a second approach where we selected genetic markers that are significantly associated with adult onset asthma in exposed subjects, but not in non-exposed subjects.

In the first approach we meta-analysed the study specific interaction effects and we selected SNPs with a fixed effect meta-analysis interaction effect with p-value <10^−4^. In the second approach we meta-analysed the genetic main effect in exposed and non-exposed subjects separately and we then selected SNPs with a genetic effect with p-value <10^−4^ only in exposed subjects based on the fixed effect model. SNPs with the same effect in exposed and non-exposed subjects were omitted by filtering on a nominal interaction effect (p-value >10^−2^).

Only SNPs present in at least two studies were included in the discovery meta-analysis, yielding to a total of 525,150 SNPs. Genome wide significance was set to a p-value < 9.5*10^−8^ based on Bonferroni correction. All SNPs selected from the discovery meta-analysis were tested for replication in an independent population, the LifeLines Cohort Study [16] (Description of study in S1 File).

To investigate if the association between genetic background, tobacco smoking and adult onset asthma was robust for the different smoking habits we assessed the genetic effects of the identified SNPs on adult onset asthma in different strata of smoking habits (ever, current and former active smoking, as well as current passive smoking) in the LifeLines cohort study: exposed versus non-exposed to ever active tobacco smoking; exposed versus non-exposed to current active tobacco smoking; exposed versus non-exposed to active smoking in the past; exposed versus non-exposed to current passive smoking (details on the exposure definitions in S1 File). The analyses were conducted using Plink 1.07 [17] and R [18]. For annotation and inspection of linkage disequilibrium (LD) patterns WGAviewer [19] was used.

## Results

**Fig 2:**
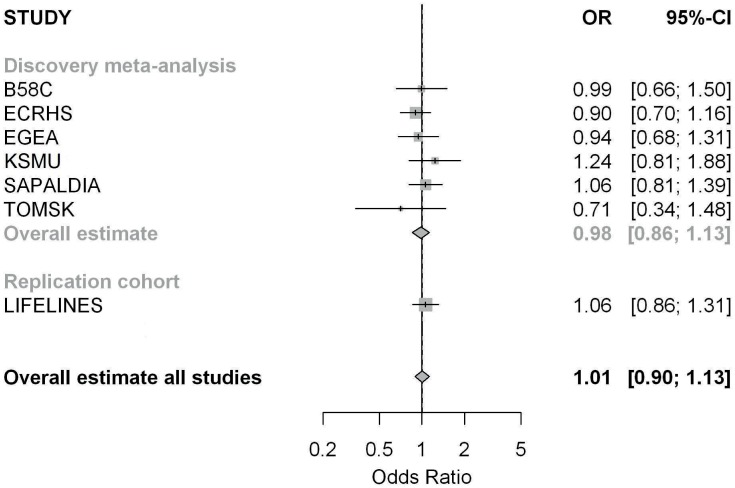
Forest plot for meta-analysis on the association between ever active tobacco smoking and adult onset asthma, without including the genetic effect.

The discovery genome-wide interaction meta-analysis consisted of 1,324 cases and 2,733 controls derived from six studies (Table 1). Overall, active tobacco smoking was not associated with adult onset asthma (Fig 2).

**Table 1:** Study populations included in GWI study on active smoking and adult onset asthma.

Study	Country	Design	Ever tobacco smokers, % (N)	N	Cases			Controls		
					Total	Exposed	(%)	Total	Exposed	(%)
*Discovery study*										
B58C	UK	Cohort	27.2 (123)	452	232	63	27.2	220	60	27.3
ECRHS	European	Multicentre	57.4 (710)	1238	353	196	55.5	885	514	58.1
EGEA	France	Cohort, family structure	49.5 (407)	822	186	90	48.4	636	317	49.8
KSMU	Russia	Case-control	64.2 (255)	397	164	110	67.1	233	145	62.2
SAPALDIA	Switzerland	Cohort	55.9 (498)	891	354	201	56.8	537	297	55.3
TOMSK	Russia	Cohort, family structure	44.4 (114)	257	35	13	37.1	222	101	45.5
TOTAL			51.9 (2107)	4057	1324	673	48.7	2733	1434	49.7
*Replication study*										
LifeLines	Netherlands	Cohort	60.1% (7496)	12475	366	225	61.5	12109	7271	60.0

Numbers are shown for subjects who were successfully genotyped and whose genotypes passed all quality checks

**Fig 3:**
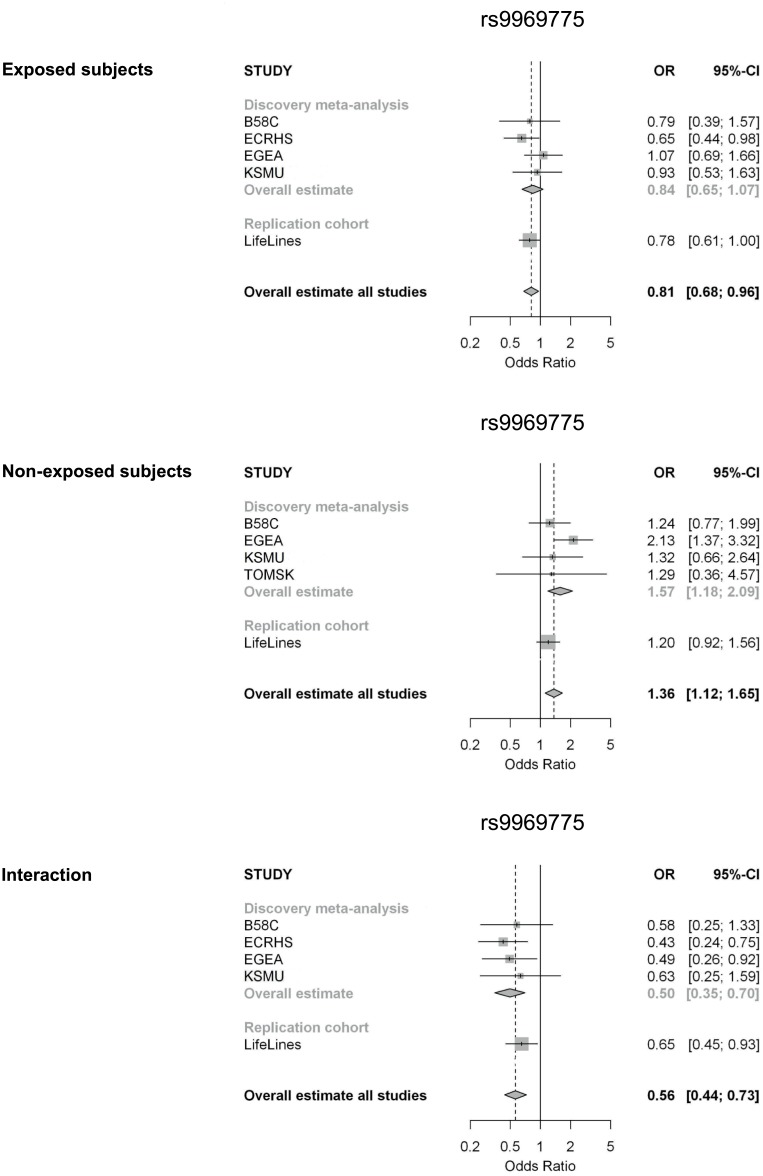
Forest plots for the meta-analysis and replication study on the genetic effect of SNP rs9969775 on chromosome 9 in subjects exposed and non-exposed to ever active tobacco smoking (identified in first approach). The bottom forest plot presents the interaction meta-analysis and replication study for this SNP. ORs are calculated using a fixed effect model.

Firstly, we identified 50 SNPs in the discovery meta-analysis with an interaction p-value<10^−4^. None of the SNPs reached genome-wide significance. The results for two SNPs showed heterogeneity across studies (p-value Q-statistic <0.05); these SNPs were omitted from further analysis. In the replication study, 29 of the 48 SNPs were included since 19 SNPs were not successfully imputed in the LifeLines Cohort Study or did not pass quality control (S1 Table). In total, 16 SNPs showed the same direction of the interaction effect in the discovery and replication analysis. None of the associations reached statistical significance in the replication study after Bonferroni correction for multiple testing for 29 SNPs (p-value<0.0017) (Table 2). One SNP reached nominal significance: rs9969775 on chromosome 9. For this SNP the interaction estimate in the discovery meta-analysis was OR_int_ = 0.50, p-value = 7.63*10^−5^ and in the replication study: OR_int_ = 0.65, p-value = 0.02 (Table 2). Fig 3 shows the forest plots with the results for the discovery studies. In the smoking stratified analysis, non-exposed subjects carrying an A allele tended to have an increased asthma risk (discovery meta-analysis OR = 1.57, p-value = 1.88*10^−3^, replication study OR = 1.20, p-value = 0.19), which was not observed in exposed subjects.

**Table 2:** Top SNPs that interact with active tobacco smoking in adult onset asthma identified in first approach (overall interaction effect)^#^.

Ch	SNP	Position	Effect allele	MAF*	Discovery meta-analysis						Replication study						Direction of the effect^¶^	
					Interaction			Exposed			Interaction			Exposed			Int	Exp
					OR_int_^§^	95%CI	P^§^	OR^§^	95%CI	P^§^	OR_int_^§^	95%CI	P^§^	OR^§^	95%CI	P^§^	D/R^¶^	D/R^¶^
1	rs4926457	245012644	C	0.42	0.65	0.52;0.80	4.49E-05	0.81	0.70;0.94	4.42E-03	1.03	0.76;1.39	0.86	1.06	0.88;1.28	0.54	-/+	-/+
1	rs10924824	244998447	G	0.42	0.64	0.52;0.79	2.96E-05	0.81	0.70;0.93	3.79E-03	0.99	0.74;1.34	0.97	1.05	0.87;1.27	0.61	-/-	-/+
1	rs4244627	245007487	G	0.42	0.64	0.52;0.79	3.52E-05	0.81	0.70;0.93	3.99E-03	1.02	0.75;1.38	0.91	1.05	0.87;1.27	0.62	-/+	-/+
1	rs10924823	244998415	T	0.42	0.65	0.52;0.80	4.28E-05	0.81	0.70;0.94	5.22E-03	0.99	0.73;1.34	0.96	1.05	0.87;1.27	0.62	-/-	-/+
2	rs1448187	111936830	T	0.28	0.65	0.53;0.81	9.34E-05	0.83	0.71;0.97	1.84E-02	1.04	0.75;1.45	0.81	1.06	0.87;1.31	0.55	-/+	-/+
2	rs2195614	221958757	A	0.43	1.51	1.23;1.85	8.10E-05	1.13	0.98;1.30	8.37E-02	1.04	0.77;1.41	0.80	1.04	0.86;1.25	0.70	+/+	+/+
2	rs2217431	221967489	A	0.43	1.53	1.24;1.88	5.14E-05	1.14	0.99;1.32	6.91E-02	1.04	0.77;1.41	0.80	1.04	0.86;1.25	0.70	+/+	+/+
2	rs13000320	237388433	C	0.17	0.57	0.44;0.76	9.00E-05	0.82	0.67;0.99	4.28E-02	0.86	0.58;1.28	0.46	0.92	0.72;1.19	0.55	-/-	-/-
3	rs428834	1629347	T	0.08	2.32	1.55;3.48	4.06E-05	1.57	1.22;2.03	5.68E-04	1.37	0.79;2.38	0.26	1.29	0.93;1.77	0.12	+/+	+/+
5	rs6863550	174552023	A	0.35	0.63	0.50;0.78	2.55E-05	0.75	0.64;0.87	1.89E-04	1.03	0.75;1.41	0.87	1.13	0.93;1.38	0.23	-/+	-/+
6	rs943801	165912238	C	0.21	0.59	0.46;0.77	6.69E-05	0.75	0.63;0.90	2.20E-03	1.00	0.70;1.42	0.99	1.13	0.91;1.41	0.27	-/0	-/+
6	rs2987296	165927063	T	0.14	0.52	0.38;0.71	3.35E-05	0.74	0.60;0.92	6.73E-03	1.14	0.76;1.70	0.53	1.12	0.87;1.43	0.37	-/+	-/+
6	rs643066	165872834	T	0.25	0.58	0.46;0.74	1.19E-05	0.79	0.67;0.94	6.77E-03	1.18	0.84;1.66	0.35	1.28	1.04;1.57	0.02	-/+	-/+
9	rs2988576	12352801	A	0.46	0.66	0.54;0.81	5.37E-05	0.75	0.65;0.87	1.13E-04	1.15	0.83;1.58	0.41	1.01	0.82;1.23	0.96	-/+	-/+
9	rs9969775	13561933	A	0.13	0.50	0.35;0.70	7.63E-05	0.84	0.65;1.07	1.50E-01	0.65	0.45;0.93	0.02	0.78	0.61;1.00	0.05	-/-	-/-
9	rs4338205	17736447	A	0.11	2.11	1.47;3.02	5.04E-05	1.46	1.16;1.84	1.18E-03	0.61	0.36;1.04	0.07	0.79	0.54;1.14	0.20	+/-	+/-
9	rs4745437	77497877	C	0.43	0.62	0.50;0.76	6.00E-06	0.82	0.71;0.95	7.69E-03	0.89	0.65;1.23	0.49	0.89	0.73;1.08	0.25	-/-	-/-
9	rs1328550	77499107	C	0.33	1.55	1.25;1.92	7.28E-05	1.21	1.05;1.41	1.04E-02	1.00	0.72;1.38	1.00	1.07	0.88;1.31	0.48	+/0	+/+
10	rs7074731	23142594	C	0.17	1.79	1.35;2.37	5.94E-05	1.32	1.10;1.59	2.93E-03	1.04	0.67;1.63	0.85	0.89	0.68;1.17	0.42	+/+	+/-
12	rs999481	5363096	G	0.42	0.66	0.53;0.81	8.26E-05	0.75	0.65;0.87	1.42E-04	0.75	0.55;1.03	0.07	0.85	0.70;1.04	0.11	-/-	-/-
12	rs1716466	118348243	G	0.41	1.52	1.24;1.87	6.66E-05	1.22	1.06;1.41	5.92E-03	0.96	0.70;1.30	0.77	1.08	0.89;1.31	0.44	+/-	+/+
12	rs7954580	128647904	A	0.14	0.55	0.41;0.74	9.99E-05	0.76	0.61;0.94	1.26E-02	0.94	0.61;1.46	0.79	0.90	0.69;1.19	0.46	-/-	-/-
13	rs9591994	58855563	C	0.33	0.64	0.52;0.80	8.09E-05	0.75	0.64;0.88	4.28E-04	0.76	0.39;1.49	0.42	1.01	0.64;1.59	0.96	-/-	-/+
13	rs9544173	75597382	G	0.08	0.40	0.27;0.59	5.67E-06	0.73	0.55;0.97	2.97E-02	0.74	0.31;1.75	0.50	0.96	0.54;1.73	0.90	-/-	-/-
16	rs8047401	79304514	T	0.35	0.66	0.53;0.81	9.98E-05	0.76	0.65;0.88	1.99E-04	0.88	0.62;1.25	0.47	0.87	0.71;1.08	0.22	-/-	-/-
17	rs11077501	66037656	C	0.36	0.65	0.53;0.80	6.60E-05	0.83	0.71;0.96	1.33E-02	0.83	0.61;1.13	0.24	1.04	0.86;1.27	0.68	-/-	-/+
20	rs1984399	40312545	A	0.42	1.59	1.29;1.96	1.31E-05	1.25	1.08;1.44	2.19E-03	0.68	0.50;0.93	0.02	0.89	0.73;1.09	0.26	+/-	+/-
20	rs727336	40318090	T	0.42	1.55	1.26;1.91	3.58E-05	1.24	1.07;1.43	3.56E-03	0.65	0.48;0.88	0.01	0.88	0.72;1.07	0.21	+/-	+/-
22	rs4553919	24941463	T	0.18	0.57	0.44;0.75	4.40E-05	0.80	0.66;0.98	2.71E-02	0.95	0.66;1.37	0.79	1.04	0.83;1.31	0.73	-/-	-/+

^#^ Selection based on interaction effect with active tobacco smoking. Additive genetic model. Interaction model included genetic effect, smoking effect, interaction effect, gender, age and informative principal components. Ch: Chromosome; OR: Odds ratio; OR_int_: Interaction Odds ratio; CI: Confidence interval; P: p-value

* MAF: Minor allele frequency (%), median of MAF in all discovery studies;

^**§**^ OR and p-value are based on fixed effect model

^**¶**^ Direction of the effect: + = positive,— = negative, 0 = no association, D/R: Discovery meta-analysis/Replication study

**Fig 4:**
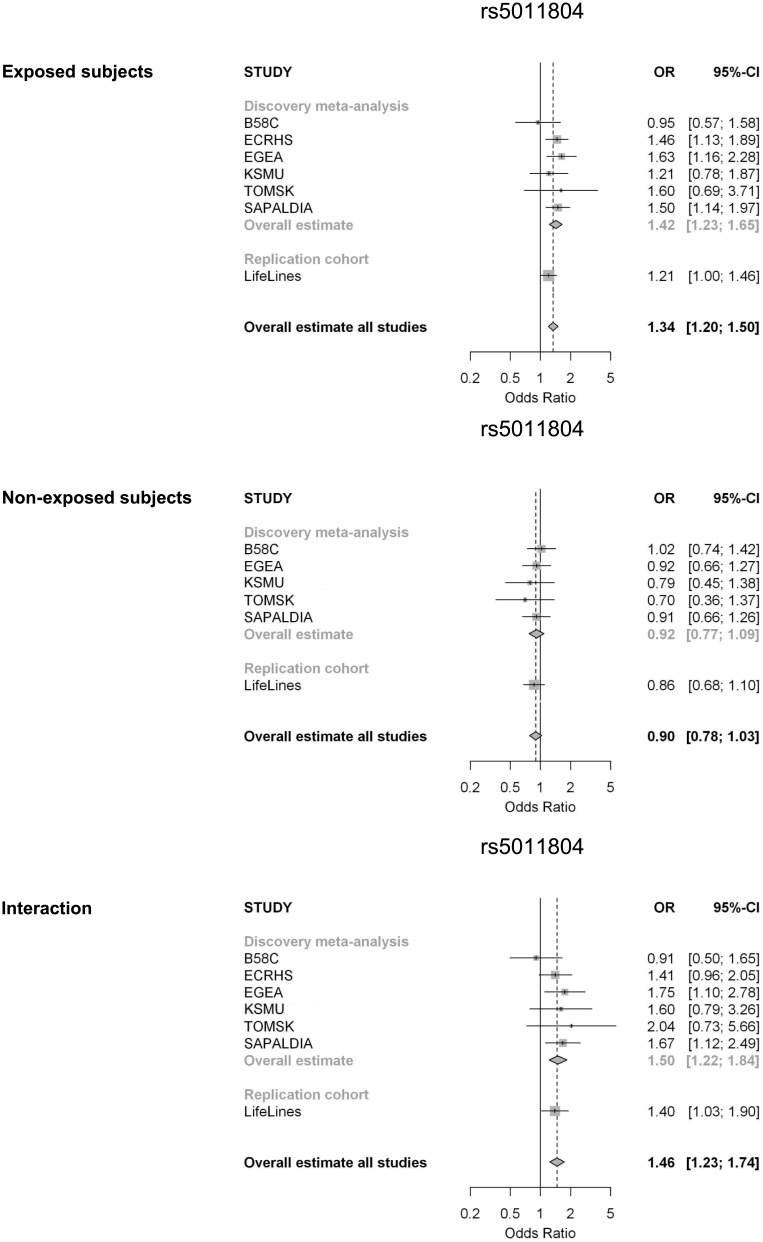
Forest plots for the meta-analysis and replication study on the genetic effect of SNP rs5011804 on chromosome 12 in subjects exposed and non-exposed to ever active tobacco smoking (identified in second approach). The bottom forest plot presents the interaction meta-analysis and replication study for this SNP. ORs are calculated using a fixed effect model.

Secondly, we identified 35 SNPs in the discovery meta-analysis with a genetic effect of p-value<10^−4^ and an interaction p-value<10^−2^. Findings did not reach genome-wide significance. None of the SNPs showed heterogeneity across studies (p-value Q-statistic <0.05). In the replication study, 27 of the 35 SNPs were included, since 8 SNPs were not successfully imputed in the LifeLines Cohort Study or did not pass quality control (S1 Table). For 15 SNPs, the direction of the effect in the exposed subjects was the same in the discovery and replication analysis. None of the associations reached statistical significance in the replication study after Bonferroni correction for multiple testing for 27 SNPs (p-value<0.0019) (Table 3). One SNP reached nominal significance in the replication: rs5011804 on chromosome 12 (OR_int_ = 1.40, p-value = 0.03). The interaction estimate for this SNP was OR_int_ = 1.50, p-value = 1.21*10^−4^ in the discovery meta-analysis (Table 3). Fig 4 shows the forest plots with results for the individual studies. In subjects who ever smoked, carriers of the minor allele C had an increased risk for asthma (discovery meta-analysis OR = 1.42, p-value = 1.56*10^−6^; replication study OR = 1.21, p-value = 0.05), while in non-exposed subjects, carriers of the C allele had no increased asthma risk (discovery meta-analysis OR = 0.92, p-value = 0.31, replication study OR = 0.86, p-value = 0.24).

**Table 3:** Top SNPs that interact with active tobacco smoking in adult onset asthma identified in second approach (genetic effect in exposed)^#^.

Ch	SNP	Position	Effect allele	MAF*	Discovery meta-analysis						Replication study						Direction of the effect^¶^	
					Interaction			Exposed			Interaction			Exposed			Int	Exp
					OR_int_^§^	95%CI	P^§^	OR^§^	95%CI	P^§^	OR_int_^§^	95%CI	P^§^	OR^§^	95%CI	P^§^	D/R^¶^	D/R^¶^
1	rs7513225	20634784	A	0.38	0.72	0.58;0.89	1.88E-03	0.74	0.64;0.86	7.88E-05	0.98	0.68;1.42	0.93	1.15	0.92;1.45	0.23	-/-	-/+
3	rs9758775	163957528	C	0.27	0.52	0.34;0.79	2.14E-03	0.48	0.34;0.67	2.31E-05	1.45	1.01;2.08	0.04	1.06	0.86;1.31	0.57	-/+	-/+
5	rs3853475	141796799	C	0.38	1.43	1.16;1.76	7.88E-04	1.36	1.18;1.57	3.68E-05	1.26	0.93;1.72	0.14	1.06	0.88;1.28	0.56	+/+	+/+
6	rs1106841	43604640	C	0.38	1.44	1.16;1.77	7.36E-04	1.35	1.17;1.56	4.17E-05	0.94	0.69;1.28	0.70	1.05	0.87;1.28	0.58	+/-	+/+
6	rs2812719	80410202	A	0.41	1.38	1.12;1.69	2.03E-03	1.38	1.20;1.59	9.39E-06	1.08	0.79;1.47	0.63	1.12	0.93;1.36	0.23	+/+	+/+
6	rs723981	80421994	T	0.07	1.80	1.24;2.63	2.22E-03	1.77	1.37;2.27	9.74E-06	0.52	0.33;0.85	0.01	0.83	0.59;1.17	0.28	+/-	+/-
6	rs1883877	80439582	A	0.07	1.86	1.27;2.71	1.36E-03	1.77	1.37;2.27	9.74E-06	0.82	0.43;1.57	0.55	0.80	0.53;1.21	0.28	+/-	+/-
7	rs2015523	88616537	T	0.16	1.51	1.12;2.02	6.11E-03	1.52	1.23;1.86	7.31E-05	1.06	0.69;1.63	0.78	0.96	0.74;1.25	0.77	+/+	+/-
8	rs7816370	3037931	A	0.17	0.68	0.51;0.90	6.18E-03	0.66	0.54;0.79	1.51E-05	1.02	0.68;1.52	0.93	0.96	0.75;1.23	0.74	-/+	-/-
8	rs17601573	87135695	C	0.54	1.37	1.11;1.68	2.67E-03	1.36	1.18;1.56	2.04E-05	1.13	0.84;1.53	0.42	1.11	0.92;1.34	0.27	+/+	+/+
9	rs2890993	14741872	G	0.15	1.66	1.23;2.25	8.94E-04	1.52	1.25;1.85	3.76E-05	1.27	0.79;2.02	0.32	1.12	0.85;1.47	0.42	+/+	+/+
9	rs17061224	77273982	T	0.13	1.47	1.10;1.97	9.79E-03	1.49	1.23;1.82	6.47E-05	1.27	0.76;2.13	0.36	1.12	0.83;1.52	0.46	+/+	+/+
10	rs7906433	3878845	T	0.24	0.64	0.50;0.83	4.30E-04	0.70	0.60;0.83	3.91E-05	1.21	0.86;1.71	0.28	1.11	0.90;1.37	0.33	-/+	-/+
11	rs3818275	35265359	C	0.34	0.66	0.53;0.82	1.87E-04	0.74	0.63;0.86	7.11E-05	1.05	0.76;1.45	0.76	1.08	0.89;1.32	0.44	-/+	-/+
12	rs11047993	25439546	A	0.46	1.45	1.18;1.78	3.86E-04	1.41	1.22;1.63	2.52E-06	1.31	0.97;1.78	0.08	1.17	0.97;1.41	0.10	+/+	+/+
12	rs11047994	25439598	A	0.40	1.43	1.17;1.77	6.70E-04	1.35	1.16;1.55	5.34E-05	1.31	0.96;1.79	0.09	1.17	0.97;1.41	0.10	+/+	+/+
12	rs4578491	25440513	A	0.46	1.47	1.19;1.80	2.64E-04	1.43	1.24;1.65	1.34E-06	1.31	0.97;1.78	0.08	1.17	0.97;1.41	0.10	+/+	+/+
12	rs5011804	25441894	C	0.46	1.50	1.22;1.84	1.21E-04	1.42	1.23;1.65	1.56E-06	1.40	1.03;1.90	0.03	1.21	1.00;1.46	0.05	+/+	+/+
13	rs4884334	58839005	G	0.32	0.70	0.56;0.87	1.78E-03	0.72	0.61;0.85	9.66E-05	1.35	0.95;1.92	0.09	1.05	0.85;1.29	0.67	-/+	-/+
17	rs8071270	66907543	T	0.29	0.67	0.53;0.84	5.28E-04	0.73	0.62;0.85	8.68E-05	0.97	0.68;1.39	0.87	0.97	0.77;1.21	0.79	-/-	-/-
17	rs7226071	66917957	G	0.29	0.67	0.54;0.85	6.61E-04	0.73	0.62;0.85	9.76E-05	0.89	0.62;1.29	0.56	0.93	0.74;1.17	0.53	-/-	-/-
17	rs6501483	66920291	G	0.29	0.67	0.54;0.85	6.74E-04	0.73	0.62;0.85	8.58E-05	0.99	0.71;1.40	0.98	0.99	0.80;1.23	0.93	-/-	-/-
17	rs2367536	66975870	C	0.29	0.72	0.57;0.90	4.76E-03	0.72	0.62;0.85	6.83E-05	1.05	0.73;1.52	0.78	0.95	0.76;1.19	0.66	-/+	-/-
18	rs724676	5916216	T	0.53	0.70	0.58;0.86	6.31E-04	0.76	0.66;0.87	9.28E-05	0.95	0.70;1.28	0.73	1.00	0.83;1.21	0.97	-/-	-/0
19	rs618940	39328412	G	0.39	0.71	0.57;0.87	9.95E-04	0.74	0.64;0.85	2.93E-05	1.33	0.97;1.83	0.07	1.23	1.02;1.49	0.03	-/+	-/+
20	rs6072658	40278039	C	0.19	0.61	0.47;0.78	1.51E-04	0.69	0.58;0.83	9.38E-05	1.61	1.10;2.35	0.01	1.20	0.96;1.49	0.11	-/+	-/+
20	rs10485689	40312475	T	0.19	0.60	0.46;0.78	1.28E-04	0.68	0.56;0.82	4.23E-05	1.40	0.98;2.00	0.07	1.17	0.95;1.46	0.15	-/+	-/+

^#^ Selection based on genetic effect in subjects exposed to active tobacco smoking. Additive genetic model. Interaction model included genetic effect, smoking effect, interaction effect, gender, age and informative principal components. Ch: Chromosome; Ref allele: Reference allele; OR: Odds ratio; OR_int_: Interaction Odds ratio; CI: Confidence interval; P: p-value

* MAF: Minor allele frequency (%), median of MAF in all discovery studies;

^**§**^ OR and p-value are based on fixed effect model

^**¶**^ Direction of the effect: + = positive,— = negative, 0 = no association, D/R: Discovery meta-analysis/Replication study

Four SNPs were identified by both approaches (Table 4), but the results for these SNPs could not be replicated in LifeLines Cohort Study. The S2 Table shows the annotation of all SNPs identified in at least one of the approaches.

**Table 4:** Top SNPs that interact with active tobacco smoking in adult onset asthma identified in both approaches^#^.

Ch	SNP	Position	Effect allele	MAF*	Discovery meta-analysis						Replication study						Direction of the effect^¶^	
					Interaction			Exposed			Interaction			Exposed			Int	Exp
					OR_int_^§^	95%CI	P^§^	OR^§^	95%CI	P^§^	OR_int_^§^	95%CI	P^§^	OR^§^	95%CI	P^§^	D/R^¶^	D/R^¶^
5	rs4912832	141632275	A	0.46	1.59	1.30; 1.96	8.04E-06	1.38	1.20;1.59	1.01E-05	1.25	0.91;1.72	0.17	1.01	0.83;1.24	0.90	+/+	+/+
5	rs4541689	141631376	G	0.46	1.61	1.31;1.98	5.40E-06	1.39	1.20;1.60	7.39E-06	1.24	0.88;1.73	0.22	0.94	0.76;1.16	0.56	+/+	+/-
19	rs1759092	39368378	G	0.40	0.65	0.53;0.80	4.99E-05	0.75	0.65;0.87	9.79E-05	1.25	0.90;1.73	0.18	1.21	0.99;1.48	0.06	-/+	-/+
20	rs7262414	40245194	A	0.19	0.59	0.46;0.77	8.63E-05	0.68	0.57;0.82	6.33E-05	1.55	1.07;2.24	0.02	1.21	0.98;1.51	0.08	-/+	-/+

^#^ Additive genetic model. Interaction model included genetic effect, smoking effect, interaction effect, gender, age and informative principal components.

Ch: Chromosome; Ref allele: Reference allele; OR: Odds ratio; OR_int_: Interaction Odds ratio; CI: Confidence interval; P: p-value

* MAF: Minor allele frequency (%), median of MAF in all discovery studies;

^**§**^ OR and p-value are based on fixed effect model

^**¶**^ Direction of the effect: + = positive, − = negative, 0 = no association, D/R: Discovery meta-analysis/Replication study

The analyses of the robustness of the results showed that the identified SNPs interacted with active tobacco smoking and not with passive smoking (Table 5), effects being particularly apparent among ex-smokers.

**Table 5:** Genetic effect of SNP rs5011804 following an additive model in the LifeLines cohort (N = 12,475), stratified by different tobacco smoke exposures.

Exposure	Stratum	N*	%	Genetic effect		
				OR	95% CI	p-value
Ever active tobacco smoking	Exposed	7496	60.1	1.21	1.00; 1.46	0.05
	Non-exposed	4979	39.9	0.86	0.68; 1.10	0.24
Current active tobacco smoking	Exposed	2800	22.5	0.84	0.61; 1.17	0.31
	Non-exposed	9666	77.5	1.14	0.97; 1.35	0.12
Ex smoker	Exposed	4624	37.1	1.44	1.14; 1.82	0.003
	Non-exposed	7842	62.9	0.89	0.73; 1.07	0.21
Current passive smoking	Exposed	2487	36.4	0.92	0.66; 1.27	0.61
	Non-exposed	4343	63.6	0.99	0.77; 1.28	0.96

* Numbers may not add up to 12,475, due to missing data on the specific exposure.

## Discussion

This study is the first hypothesis-free genome-wide study specifically aiming to identify SNPs that interact with active tobacco smoking with respect to asthma onset at adult age. The results are based on data from GABRIEL, a large consortium on adult onset asthma. We found suggestive evidence for an interaction between active tobacco smoking and rs9969775 on chromosome 9 and rs5011804 on chromosome 12. Both SNPs are intergenic markers that do not annotate to genes nor do SNPs in LD with these markers.

The SNPs found have not been identified previously in general GWA studies on asthma. Although the identified markers do not annotate for a protein coding region, they may have a regulatory function. rs9969775 is a tri-allellic polymorphism but in our datasets only two alleles were present (effect allele: A, reference allele: C). Rs9969775 is located between the *FLJ41200* gene (distance ~ 129 KB, also known as LINC01235) and *RP11-284P20*.*1* (distance ~ 366 KB). Both *FLJ41200* and *RP11-284P20*.*1* are long intergenic non-protein coding RNA genes. With the development of whole genome and transcriptome sequencing technologies, long noncoding RNAs have received increased attention. Multiple studies indicate that they can regulate gene expression in many ways, including chromatin modification, transcription and post-transcriptional processing [20]. A search for rs9969775 in the ENCODE database (using the WashU Epi Genome Browser http://epigenomegateway.wustl.edu/) showed that this SNP is located at a CpG site with a high methylation score in lung tissue. Further analysis of this SNP using Haploreg indicated that this SNP is located in a region of active chromatin in the lung, as indicated by a DNASE I hypersensitivity site, in an enhancer region (Haploreg version 4.1: http://archive.broadinstitute.org/mammals/haploreg/haploreg.php).

The second identified SNP, rs5011804, is located between the *KRAS* gene (distance ~ 38 KB) and the *RPL39P27* gene (distance ~ 120 KB). The *KRAS* gene encodes a protein that is a member of the small GTPase superfamily. Small GTPases regulate a wide variety of processes in the cell, including growth, cellular differentiation, cell movement and lipid vesicle transport. *RPL39P27* is a ribosomal protein pseudogene. Pseudogenes are fragments of genes that were functional but have been silenced by one or more mutations[21]. It was assumed that pseudogenes were not functional but recent studies suggest that they may have a functional role such as gene expression, gene regulation, and generation of genetic diversity [22]. Finally, to gain more insight in the possible regulatory roles of rs9969775 and rs5011804 on gene expression, data from the Genotype-Tissue Expression project (http://www.gtexportal.org/home/) was used. The results showed that the SNPs were not associated with gene expression of any gene in any tissue. In summary, our identified SNPs are located in regions with potential regulatory function and future research is needed to unravel their role in adult asthma further. Of interest, the two SNPs that were previously reported to be associated with adult onset asthma [15] (rs17843604 and rs9273349 on chromosome 6) showed nominal significant associations with asthma in both smokers and non-smokers but no interaction with active tobacco smoking in our meta-analysis (S3 Table).

The GWI study design is specifically suited to identify novel SNPs that interact with an environmental exposure in an unbiased way. Genes identified to interact with active tobacco smoking are crucial for further insight in the etiology of adult onset asthma and development of effective strategies for asthma prevention. A strength of our study is that we followed two different approaches to detect SNPs that show a differential effect in subjects exposed and non-exposed to smoking. The classical GWI study approach is to select SNPs with the largest interaction effect. Since we also aimed to identify subpopulations that are genetically susceptible for active tobacco smoking we followed a second approach in which we selected SNPs that only affected the risk of asthma in exposed subjects and not in non-exposed subjects. In our analyses, four SNPs were identified with both approaches.

Since adult onset asthma is not common, only a subset of asthmatics is exposed, and the expected effect size is small, a large sample size is needed to obtain a genome-wide significant finding. In this study we combined data from multiple studies to achieve this. We additionally harmonized the exposure and outcome definitions in the different studies as much as possible to improve the chance of finding significant interactive effects. However, small differences in these definitions between studies could create random error which compromises study power and thus makes it harder to detect a significant interaction [23].

A limitation of our study is that active tobacco smoking is related to exposure to environmental smoke at different periods in life, which makes it difficult to disentangle the effects of these exposures. Therefore, we assessed the genetic effects of the identified SNPs on adult onset asthma in different strata of smoking habits in the LifeLines Cohort Study. Results showed that genetic effects of the identified SNPs were particularly apparent among ex smokers.

Two studies included in the meta-analysis contained cross-sectional and retrospectively collected data. In these studies, asthma onset before the start of smoking could not be ruled out. Inclusion of these subjects would lead to a dilution of the actual interaction between genetics and ever smoking on adult onset asthma. Since data from the LifeLines Cohort Study showed that only eight (3.6%) subjects out of 225 ever smoking adult onset asthmatics started smoking after the start of adult onset asthma (data not shown), it is unlikely that this issue biased our results.

A general problem in GWI studies is their limited power, due to often a small number of subjects with overlapping exposures and genotypes [24,25]. The power to detect an interaction can be increased by assessing the association between exposure and genotype in a case-only design or a two-step design [24,25] A case-only design assumes that exposure and genotype are independent. We chose not to use this design given the known strong genetic component of smoking addiction, and relatively modest violations of this assumption can have a substantial impact on bias relating to the interaction parameters [26], hence leading to false positive or false negative findings [27]. In a two-step design the interaction is tested among a selection of SNPs. The method we used to detect interactions between exposure and genotype did not assume exposure and genotype independence nor did we a priori select SNPs. To limit the possibility to miss possible interaction effects, we first selected the most promising SNPs using an arbitrary threshold for interaction (p <10^−4^) and included them in a replication study. A similar approach has been used successfully in a GWI study on interaction between genetic markers and waist hip ratio on total serum cholesterol [28].

In summary, we performed two approaches for GWI analyses and identified SNPs on chromosome 9 and 12, both intergenic variants with potential regulatory functions. These are novel SNPs, previously unidentified by regular genome-wide association and candidate gene studies that showed suggestive evidence for interaction with active tobacco smoking in adult onset asthma. We propose that future studies replicate our findings.

## Supporting information

S1 File Description of individual studies. (DOC)

S1 Table Complete results of all identified SNPs. (XLS)

S2 Table Annotation of the top SNPs identified in both approaches. (DOC)

S3 Table Results for rs17843604 and rs9273349. (XLS)

S1 Checklist PRISMA 2009 checklist. (DOC)

S2 Checklist Meta-analysis on genetic association studies checklist | PLOS ONE. (DOCX)
