# Supplementary material for: Adult onset asthma and interaction between genes and active tobacco smoking: The GABRIEL consortium
Source: PLoS One. 2017 Mar 2;12(3):e0172716. doi: 10.1371/journal.pone.0172716 (PMC5333809; doi:10.1371/journal.pone.0172716)
Supplement: S1 File — (DOC) [file pone.0172716.s001.doc]

Supporting information

Adult onset asthma and interaction between genes and active tobacco smoking:
the GABRIEL consortium.

Vonk et al.

**Description of individual studies**

British 1958 birth cohort (B58C)

The 1958 British birth cohort is an ongoing follow-up of persons born in Britain during one week in 1958 (<http://www.b58cgene.sgul.ac.uk/>). At age 44-45 years, a full biomedical examination was performed from which DNA samples were prepared for use as a nationally representative reference series for genetic case-control studies.^1^ About half of the cohort members with a history of asthma ascertained at any age up to 42 years, and a similar number of non-asthmatic controls, were included in the GABRIEL meta-analysis^2^. For the purpose of this interaction analysis, adulthood asthmatics were defined as persons reporting asthma ever at any follow-up from 16 years of age. Controls were defined with exclusion of childhood onset case. Ever active smoking was ascertained by self-report and interview at age 23, 33 and 42 years.

ECRHS

Sixteen centres (eight countries) in the European Community Respiratory Health Survey (ECRHS) have contributed samples to GABRIEL ([http://www.ecrhs.org](http://www.ecrhs.org/)).^3,4^ In each centre, a representative community-based sample of at least 3000 adults aged 20-44 years were invited to complete a brief postal questionnaire asking about respiratory symptoms (ECRHS I - Stage 1) between 1991-1993. A random sample of these (600 per centre) underwent intensive further investigation (ECRHS I - Stage 2 – random sample). Participants who had symptoms highly suggestive of asthma but who had not been selected at random to take part in Stage 2, were also invited to undergo intensive investigations (ECRHS I - Stage 2- enriched sample). About ten years later all adults who had taken part in Stage 2 were recontacted (ECRHS II) and again asked about respiratory symptoms. Samples suitable for DNA extraction were collected. For the GABRIEL initiative all cases of asthma were identified (participants from the random or enriched sample who said yes to the question „Have you ever had asthma? at either ECRHS I or ECRHS II). Adulthood onset asthma cases were defined as asthma from 16 years of age. Active smoking was defined as “Ever active smoking by anyone from 16 years of age”.

EGEA

EGEA is a 12-year longitudinal survey which combines a case-control study and a family study (https://egeanet.vjf.inserm.fr/). The first survey (EGEA1) took place between 1992 and 1995.^5^ The study population included 388 asthmatics recruited in chest clinics and their 1,244 family members plus 415 population-based controls (total of 2,047 subjects). The probands (asthmatics and controls) were between 7 and 70 years old at time of study. All probands and their two parents were of European ancestry and were born in France. The second survey (EGEA2) was conducted between 2003 and 2007 and included follow-up data in 1,543 subjects from the initial cohort and 73 new family members.^6^ Data collected through face-to-face interviews and examination included extensive phenotypic characterization (detailed clinical data based on standardized questionnaire, skin prick tests, lung function tests, bronchial responsiveness, blood samples, white blood cell counts, total IgE), data on risk factors (environmental exposures, diet, physical activity, hormone-related events) and drug consumption. The study protocol was approved by the institutional ethics committee (baseline study: Cochin Port-Royal Hospital, Paris; follow-up: Necker-Enfants Malades Hospital, Paris), and all participants gave written informed consent. Asthma was defined with a positive answer to “Have you ever had attacks of breathlessness at rest with wheezing?” or “Have you ever had asthma attacks?” or being recruited as an asthma case in chest clinics. Adulthood asthma was defined as asthma onset from the age of 16 years. Controls were defined as subjects without asthma. Ever active smoking was defined as an affirmative answer on the question: "Do you smoke or have you ever smoked one cigarette per day or more for as long as a year? ".

Kursk State Medical University (KSMU)

KSMU is a population-based case-control study of adult cases of asthma and controls matched for age and sex.^7^ A total of 429 unrelated subjects were recruited in this study, (215 patients with asthma and 214 controls). The study subjects were of Russian origin from Central Russia. All patients were recruited at the Department of Pulmonology, Kursk Regional Clinical Hospital between 2003 and 2004. Additional adult patients with asthma and healthy subjects (>200 samples) from the same population were recruited between 2007 and 2008 specially in order to increase final sample size for the GWAS initiative. All patients were diagnosed with asthma by the presence of characteristic symptoms, reversibility of airway obstruction or airway hyperresponsiveness to methacholine. All control subjects were enrolled in accordance with the following criteria: (1) no symptoms and history of allergic diseases, (2) normal total serum IgE levels, (3) and normal pulmonary function test results. Childhood onset asthma cases were excluded from the analyses. Personal data, including smoking status and age of the disease onset, was collected through in-person interviews. Active smoking was defined as ever active smoking. The study was approved by the Ethical Review Committee of Kursk State Medical University, and the subjects who were recruited gave informed consent. After QC a total of 568 subjects were retained in the GWAS analyses.

SAPALDIA

SAPALDIA is a population-based cohort that originally recruited subjects aged 18 to 60 from population registries in eight Swiss communities.^8^ Subjects were obtained from among 6,055 SAPALDIA cohort subjects that participated in both, the baseline (1991) and follow-up (2002) examinations and agreed to providing blood for genetic analysis. At both baseline and follow-up examination subjects underwent spirometry as well as a detailed interview on respiratory health, smoking history and lifestyle factors. At follow-up examination, 8,047 of 9,651 baseline subjects re-participated in at least one part of the study and a formal biobank was established. SAPALDIA questions about smoking and asthma status were equivalent to those used by the ECRHS. Asthma status was defined by an affirmative answer to the question “Have you ever had asthma” at baseline and/or follow-up interview. Adulthood onset was defined as onset from 16 years of age. Controls were defined as subjects who never had asthma in their lifetime. A random sample was drawn from all controls with available GWAS data to match the proportional distribution of childhood and adulthood onset asthma. Ever active tobacco smoking was defined based on an affirmative answer to the question, whether the participant actively smoked or had been smoking in either of the surveys.

TOMSK

TOMSK is a population-based family study conducted by the Research Institute of Medical Genetics and Siberian State Medical University (Tomsk, Russia) from 1998 onwards.^9^ Both nuclear families and extended pedigrees were recruited through atopic bronchial asthmatic probands. All participants were Russians or of a mixed ethnic origin due to marriages between Russians and major East Slavonic populations (Ukrainians, Byelorussians). Altogether, 196 families were studied, out of which 150 families were recruited in Tomsk Region Children Hospital and Tomsk Region Hospital (Tomsk, Russia), and 46 families were recruited in the city of Irkutsk hospitals by the staff of the Irkutsk State Institute of Doctors Advanced Training (Irkutsk, Russia). Both probands and their relatives were clinically examined to establish diagnosis of asthma and atopy by the GINA criteria (Global Initiative for Asthma: Global Strategy for Asthma Management and Prevention. http://www.ginasthma.org). Besides the clinical examination, laboratory and functional testing were conducted to assess common IgE levels (solid-phase immune-enzyme assay), specific sensitization (skin-prick tests), lung volumes (spirometry), and airway responsiveness (bronchoprovocative tests with methacholine). Controls were defined with exclusion of childhood onset case. Active smoking was defined as ever active smoking.

LifeLines Cohort Study

The LifeLines Cohort Study is a three-generation cohort that is designed to investigate universal risk factors and their modifiers for multifactorial diseases.^10^ It is an observational follow-up study in a large representative sample of the population of the northern provinces of the Netherlands. Firstly, a random sample of persons aged between 25 and 50 years are contacted through their general practitioner and are invited to participate. Subsequently these probands invite their family members if present to take part as well (parents, partner, parents in law, children), resulting in a three-generation study. At enrollment subjects undergo a medical examination where blood sample is collected for DNA extraction. Participants filled in a questionnaire at baseline containing a question on whether they ever had asthma, whether the diagnosis has been confirmed by a physician and what was the age of onset. Adulthood onset asthma was defined as asthma onset from the age of 16 years. Ever active smoking was defined as any smoking at any age, starting before the onset of asthma. Current active tobacco smoking was defined as an affirmative answer on the question: “Do you smoke or have your smoked in the past month?”. Current passive smoking was defined as” “Were you exposed regularly in the past 12 months to passive tobacco smoking?” Childhood onset cases were excluded from the analyses. Genotyping of 301,232 SNPs was performed with using Illumina HumanCytoSNP-12v2 array. Samples for 13,301 individuals were genotyped and passed QC. The data was imputed by BEAGLE 3.0.

**Local Medical Ethical Review Committees**

B58C South East England Multi-Centre Research Ethics Committee and the National Research Ethics Service, London & South East Committee

ECRHS NRES Committee London - Stanmore

EGEA Institutional ethics committees of Cochin Port-Royal Hospital and Necker-Enfants Malades Hospital, Paris.

KSMU Ethical Review Committee of Kursk State Medical University (KSMU)

SAPALDIA Swiss Academy of Medical Sciences and ethics committees of all regional study sites

(current appropriate cantonal ethics committees names are: Ethikkommission Nordwest- und Zentralschweiz, Commission cantonale d'éthique de la recherche de Genève, Kantonale Ethikkommission Zürich, Commission cantonale d'éthique de la recherche sur l'être humain, Comitato etico cantonale.)

TOMSK Ethics Committees of the Research Institute for Medical Genetics and Siberian State Medical University, Tomsk (Russian Federation).

LifeLines Medical ethical committee of the University Medical Center Groningen

**Supplementary Methods**

**Genotyping and quality control**

Genotyping of the GABRIEL study was performed using the Illumina Human610 quad array (www.illumina.com) at CEA-Centre National de Génotypage, Evry, France. Samples from cases and controls were randomly distributed on 96-well plates. Family relationships were confirmed or revised based on the results of an identity-by-state (IBS) analysis. An ancestry analysis was carried out using the EIGENSTRAT2.0 software and putative non-European samples were excluded from the analyses. The analyses were restricted to SNPs fulfilling the following quality control criteria: (1) genotype missing rate <3% in both cases and controls; (2) minor allele frequency ≥ 1% in controls; (3) consistency with Hardy-Weinberg equilibrium in controls (P>0.0001). Informative principal components for within-Europe diversity were included as covariates in the association analysis.

Genotyping of the LifeLines study was performed with using Illumina HumanCytoSNP-12v2 array ([www.illumina.com](http://www.illumina.com/)) at the Genotyping laboratory of the University Medical Center Groningen. Quality controls of the data are based on SNP filtering on minor allele frequency (MAF) above 0.01, Hardy-Weinberg equilibrium (HWE) P-value >0.0001, call rate of 0.95 and principal component analysis (PCA) to check for population outliers. Only unrelated and Caucasian-ancestry samples were included in the analyses.

Acknowledgements

The GABRIEL study (a multidisciplinary study to identify the genetic and environmental causes of asthma in the European Community) was supported by the European Commission, contract number 018996 under the Integrated Program LSH-2004-1.2.5-1.

EGEA

We thank the EGEA cooperative group: **Coordination:** V Siroux (epidemiology, PI since 2013); F Demenais (genetics); I Pin (clinical aspects); R Nadif (biology); F Kauffmann (PI 1992-2012). **Respiratory epidemiology:** Inserm U 700, Paris: M Korobaeff (Egea1), F Neukirch (Egea1); Inserm U 707, Paris: I Annesi-Maesano (Egea1-2); Inserm CESP/U 1018, Villejuif: F Kauffmann, N Le Moual, R Nadif, MP Oryszczyn (Egea1-2), R Varraso; Inserm U 823, Grenoble: V Siroux. **Genetics:** Inserm U 393, Paris: J Feingold; Inserm U 946, Paris: E Bouzigon, F Demenais, MH Dizier; CNG, Evry: I Gut (now CNAG, Barcelona, Spain), M Lathrop (now Univ McGill, Montreal, Canada). **Clinical centers:** Grenoble: I Pin, C Pison; Lyon: D Ecochard (Egea1), F Gormand, Y Pacheco; Marseille: D Charpin (Egea1), D Vervloet (Egea1-2); Montpellier: J Bousquet; Paris Cochin: A Lockhart (Egea1), R Matran (now in Lille); Paris Necker: E Paty (Egea1-2), P Scheinmann (Egea1-2); Paris-Trousseau: A Grimfeld (Egea1-2), J Just. **Data and quality management:** Inserm ex-U155 (Egea1): J Hochez; Inserm CESP/U 1018, Villejuif: N Le Moual; Inserm ex-U780: C Ravault (Egea1-2); Inserm ex-U794: N Chateigner (Egea1-2); Grenoble: J Quentin-Ferran (Egea1-2). The authors thank all those who participated to the setting of the study and on the various aspects of the examinations involved: interviewers, technicians for lung function testing, coders, those involved in quality control, data management and all those who supervised the study in all centers. The authors are grateful to the three CIC-Inserm of Necker, Grenoble and Marseille who supported the study and in which subjects were examined. They are indebted to all the individuals who participated without whom that study would not have been possible.

**EGEA sources of funding:** INSERM-Ministry of Research 'Cohortes et Collections' grant (4CH06G). French Ministry of Higher Education and Research, University Paris Diderot-Paris 7, grants from the French Agency for Environmental and Occupational Health Safety (grant AFSSETAPR- SE-2004), the French National Agency for Research (grants ANR-05-SEST-020-02/05-9-97, ANR-06-CEBS and ANR-11-BSV1-027-GWIS-AM), PHRC-Paris, Merck Sharp & Dohme (MSD)), the “Fonds de Dotation Recherche en Santé Respiratoire” (FRSR-AO-2011), Région Ile-de-France (DIM-SEnT 2011). For the genotyping (as for all Gabriel groups): The GABRIEL genotyping was supported by a contract from the European Commission Framework Programme 6 (018996) and grants from the French Ministry of Research.

Kursk State Medical University (KSMU)

The KSMU team is supported by the Federal Targeted Program “Scientific and Scientific-Pedagogical Personnel of the Innovative Russia“ of the Ministry of Education and Science of Russian Federation. The study was supported in part by a grant from the President of Russian Federation of Federal Agency for Science and Innovation (MD-3571.2008.7). The authors thank Drs Mikhail Kozhuhov and Valery Panfilov from the Kursk Regional Clinical Hospital for their assistance in assembling patients with asthma, as well as for their help in diagnosis of the disease.

SAPALDIA

Research support: the Swiss National Science Foundation (grants no 33CSCO-108796, 3247BO-104283, 3247BO-104288, 3247BO-104284, 3247-065896, 3100-059302, 3200-052720, 3200-042532, 4026-028099), the Federal Office for Forest, Environment and Landscape, the Federal Office of Public Health, the Federal Office of Roads and Transport, the canton's government of Aargau, Basel-Stadt, Basel-Land, Geneva, Luzern, Ticino, Valais, and Zürich, the Swiss Lung League, the canton's Lung League of Basel Stadt/ Basel Landschaft, Geneva, Ticino, Valais and Zurich, SUVA, Freiwillige Akademische Gesellschaft, UBS Wealth Foundation, Talecris Biotherapeutics GmbH, Abbott Diagnostics, European Commission 018996 (GABRIEL), Wellcome Trust WT 084703MA. None of the funders had any role in study design, data collection and analysis, decision to publish, or preparation of the manuscript.

Current SAPALDIA Team: Study directorate: T Rochat (p), NM Probst Hensch (e/g), JM Gaspoz (c), N Künzli (e/exp), C Schindler (s). Scientific team: JC Barthélémy (c), W Berger (g), R Bettschart (p), A Bircher (a), O Brändli (p), C Brombach (n), M Brutsche (p), L Burdet (p), M Frey (p), U Frey (pd), MW Gerbase (p), D Gold (e/c/p), E de Groot (c), W Karrer (p), R Keller (p), B Martin (pa), D Miedinger (o), U Neu (exp), L Nicod (p), M Pons (p), F Roche (c), T Rothe (p), E Russi (p), P Schmid-Grendelmeyer (a), A Schmidt-Trucksäss (pa), A Turk (p), J Schwartz (e), D. Stolz (p), P Straehl (exp), JM Tschopp (p), A von Eckardstein (cc), E Zemp Stutz (e). Scientific team at coordinating centers: M Adam (e/g), E Boes (g), PO Bridevaux (p), D Carballo (c), E Corradi (e), I Curjuric (e), J Dratva (e), A Di Pasquale (s), E Dupuis Lozeron (s), M Germond (s), L Grize (s), D Keidel (s), S Kriemler (pa), A Kumar (g), M Imboden (g), N Maire (s), A Mehta (e), H Phuleria (exp), E Schaffner (s), GA Thun (g) A Ineichen (exp), M Ragettli (e), M Ritter (exp), T Schikowski (e), M Tarantino (s), M Tsai (e), M Wanner (pa) (a) allergology, (c) cardiology, (cc) clinical chemistry, (e) epidemiology, (exp) exposure, (g) genetic and molecular biology, (m) meteorology, (n) nutrition, (o) occupational health, (p) pneumology, (pa) physical activity, (pd) pediatrics, (s) statistics

Acknowledgements: The study could not have been done without the help of the study participants, technical and administrative support and the medical teams and field workers at the local study sites. Local fieldworkers : Aarau: S Brun, G Giger, M Sperisen, M Stahel, Basel: C Bürli, C Dahler, N Oertli, I Harreh, F Karrer, G Novicic, N Wyttenbacher, Davos: A Saner, P Senn, R Winzeler, Geneva: F Bonfils, B Blicharz, C Landolt, J Rochat, Lugano: S Boccia, E Gehrig, MT Mandia, G Solari, B Viscardi, Montana: AP Bieri, C Darioly, M Maire, Payerne: F Ding, P Danieli A Vonnez, Wald: D Bodmer, E Hochstrasser, R Kunz, C Meier, J Rakic, U Schafroth, A Walder. Administrative staff: C Gabriel, R Gutknecht.

LifeLines Cohort Study

The LifeLines Cohort Study, and generation and management of GWAS genotype data for the LifeLines Cohort Study is supported by the Netherlands Organization of Scientific Research NWO (grant 175.010.2007.006), the Economic Structure Enhancing Fund (FES) of the Dutch government, the Ministry of Economic Affairs, the Ministry of Education, Culture and Science, the Ministry for Health, Welfare and Sports, the Northern Netherlands Collaboration of Provinces (SNN),  the Province of Groningen, University Medical Center Groningen, the University of Groningen, Dutch Kidney Foundation and Dutch Diabetes Research Foundation. The authors are grateful to the study participants, the staff from the LifeLines Cohort Study and Medical Biobank Northern Netherlands,and the participating general practitioners.

References

1.     Strachan DP, Rudnicka AR, Power C, Shepherd P, Fuller E, Davis A, et al. Lifecourse influences on health among British adults: effects of region of residence in childhood and adulthood. Int J Epidemiol 2007; 36(3): 522-31.

2.     Moffatt MF, Gut IG, Demenais F, Strachan DP, Bouzigon E, Heath S, et al. A large-scale, consortium-based genomewide association study of asthma. N Engl J Med 2010; 363(13): 1211-21.

3.     Burney PG, Luczynska C, Chinn S, Jarvis D. The European Community Respiratory Health Survey. Eur Respir J 1994; 7(5): 954-60.

4.     The European Community Respiratory Health Survey II. Eur.Respir.J. 2002; 20(5): 1071-9.

5.     Kauffmann F, Dizier MH, Annesi-Maesano I, Bousquet J, Charpin D, Demenais F, et al. EGEA (Epidemiological study on the Genetics and Environment of Asthma, bronchial hyperresponsiveness and atopy)-- descriptive characteristics. Clin Exp Allergy 1999; 29 Suppl 4: 17-21.

6.     Siroux V, Boudier A, Bousquet J, Bresson JL, Cracowski JL, Ferran J, et al. Phenotypic determinants of uncontrolled asthma. J Allergy Clin Immunol 2009; 124(4): 681,7.e3.

7. Polonikov A.V., Ivanov V.P., Solodilova M.A., Kozhuhov M.A., Panfilov V.I. Tobacco smoking, fruit and vegetable intake modify association between -21A>T polymorphism of catalase gene and risk of bronchial asthma. Journal of Asthma. 2009; 46(3): P.217-224.

8. Medea Imboden, Emmanuelle Bouzigon, Ivan Curjuric, Adaikalavan Ramasamy, Ashish Kumar, Dana B. Hancock Genome-wide association study of lung function decline in adults with and without asthma. J Allergy Clin Immunol 2012; 129(5): 1218–1228

9.     Freidin MB, Kobyakova OS, Ogorodova LM, Puzyrev VP. Association of polymorphisms in the human IL4 and IL5 genes with atopic bronchial asthma and severity of the disease. Comp Funct Genomics 2003; 4(3): 346-50.

10.     Stolk RP, Rosmalen JG, Postma DS, de Boer RA, Navis G, Slaets JP, et al. Universal risk factors for multifactorial diseases: LifeLines: a three-generation population-based study. Eur J Epidemiol 2008; 23(1): 67-74.
