# Supplementary material for: Adult onset asthma and interaction between genes and active tobacco smoking: The GABRIEL consortium
Source: PLoS One. 2017 Mar 2;12(3):e0172716. doi: 10.1371/journal.pone.0172716 (PMC5333809; doi:10.1371/journal.pone.0172716)
Supplement: S2 Table — (DOC) [file pone.0172716.s003.doc]

S2 Table. Annotation of the top SNPs identified in both approaches

| **Ch** | **SNP** | **Effect allele** | **Closest gene*** | **Type** |
| --- | --- | --- | --- | --- |
| Identified in approach 1: meta-analysis of overall interaction effect | | | |  |
| 1 | rs7513225 | A |  | 5KB_upstream_variant |
| 3 | rs9758775 | C |  | intergenic_variant |
| 5 | rs3853475 | C |  | nc_transcript_variant; |
| 6 | rs1106841 | C | XPO5 | synonymous_codon;NMD_t |
| 6 | rs2812719 | A | SH3BGRL2 | intron_variant |
| 6 | rs723981 | T | SH3BGRL3 | intron_variant |
| 6 | rs1883877 | A | SH3BGRL4 | intron_variant |
| 7 | rs2015523 | T | ZNF804B | intron_variant |
| 8 | rs7816370 | A | CSMD1 | nc_transcript_variant; |
| 8 | rs17601573 | C | PSK | intron_variant |
| 9 | rs2890993 | G | FREM1 | nc_transcript_variant; |
| 9 | rs17061224 | T |  |  |
| 10 | rs7906433 | T |  | intergenic_variant |
| 11 | rs3818275 | C | SLC1A2 | intron_variant;5KB_dow |
| 12 | rs11047993 | A | U4 | intergenic_variant |
| 12 | rs11047994 | A | U4 | intergenic_variant |
| 12 | rs4578491 | A | U4 | intergenic_variant |
| 12 | rs5011804 | C | U4 =spliceosomal RNA  RPL39P27 = ribosomal protein L39 pseudogene 27 | intergenic_variant |
| 13 | rs4884334 | G | U7 | intergenic_variant |
| 17 | rs8071270 | T | U6 | intergenic_variant |
| 17 | rs7226071 | G | U6 | intergenic_variant |
| 17 | rs6501483 | G | U6 | intergenic_variant |
| 17 | rs2367536 | C |  | intergenic_variant |
| 18 | rs724676 | T | L3MBTL4 | intergenic_variant |
| 19 | rs618940 | G | LSM14A | intergenic_variant |
| 20 | rs6072658 | C | PTPRT | intron_variant |
| 20 | rs10485689 | T | PTPRT | intron_variant |
|  |  |  |  |  |
| Identified in approach 2: meta-analysis of overall genetic effect in exposed | | | | |
| 1 | rs4926457 | C |  | nc_transcript_variant; |
| 1 | rs10924824 | G | SCCPDH | 500B_downstream_varian |
| 1 | rs4244627 | G |  | nc_transcript_variant; |
| 1 | rs10924823 | T | SCCPDH | 500B_downstream_varian |
| 2 | rs1448187 | T |  | nc_transcript_variant; |
| 2 | rs2195614 | A |  | intergenic_variant |
| 2 | rs2217431 | A |  | intergenic_variant |
| 2 | rs13000320 | C |  | intergenic_variant |
| 3 | rs428834 | T |  | intergenic_variant |
| 5 | rs6863550 | A |  | intergenic_variant |
| 6 | rs943801 | C | PDE10A;PDE10 | intron_variant;5KB_ups |
| 6 | rs2987296 | T | PDE10A;PDE10 | intron_variant |
| 6 | rs643066 | T | PDE10A;PDE11 | intron_variant |
| 9 | rs2988576 | A | U2 | intergenic_variant |
| 9 | rs9969775 | A |  | intergenic_variant |
| 9 | rs4338205 | A | SH3GL2 | intron_variant |
| 9 | rs4745437 | C |  | intergenic_variant |
| 9 | rs1328550 | C |  | intergenic_variant |
| 10 | rs7074731 | C | U6 | intergenic_variant |
| 12 | rs999481 | G |  | 500B_downstream_varian |
| 12 | rs1716466 | G |  | nc_transcript_variant; |
| 12 | rs7954580 | A | TMEM132D | intron_variant |
| 13 | rs9591994 | C | U7 | intergenic_variant |
| 13 | rs9544173 | G |  | intergenic_variant |
| 16 | rs8047401 | T | CDY | nc_transcript_variant; |
| 17 | rs11077501 | C |  | intergenic_variant |
| 20 | rs1984399 | A | PTPRT | intron_variant |
| 20 | rs727336 | T | PTPRT | intron_variant |
| 22 | rs4553919 | T | SEX6L | intron_variant |
|  |  |  |  |  |
| Identified in approach 1 and 2: meta-analysis of overall interaction effect | | | |  |
| 5 | rs4912832 | A |  | intergenic_variant |
| 5 | rs4541689 | G |  | intergenic_variant |
| 19 | rs1759092 | G | LSM14A | intron_variant |
| 20 | rs7262414 | A | PTPRT | intron_variant |

* Closest gene within range of 500kb of the position of the SNP.
