# Supplementary material for: Adult onset asthma and interaction between genes and active tobacco smoking: The GABRIEL consortium
Source: PLoS One. 2017 Mar 2;12(3):e0172716. doi: 10.1371/journal.pone.0172716 (PMC5333809; doi:10.1371/journal.pone.0172716)
Supplement: S2 Checklist — (DOCX) [file pone.0172716.s006.docx]

**Meta-analysis on Genetic Association Studies Checklist | PLOS ONE**

|  | Item | Section name and paragraph number within manuscript |
| --- | --- | --- |
|  | **Introduction** |  |
| 1 | Provide a detailed justification for the polymorphism studied; if a single polymorphism was analyzed, give details as to why others were not included in the meta-analysis. | NA- no candidate gene study or other SNP selection. All available SNPs were studied in a GWA study |
| 2 | Provide a detailed justification for the population(s) and clinical condition studied. | Methods - Subjects  Introduction |
|  | **Methods** |  |
| 3 | Provide full details of the search strategy employed; outline the full electronic search strategy –specific combination of keywords and any limits applied- for at least one database. Specify whether synonyms of polymorphisms/genes (e.g. SNP number) were searched. | NA - The study is not a systematic review but includes all studies with data on adult onset asthma within the Gabriel Consortium |
| 4 | Report full details on the inclusion and exclusion criteria applied for selecting studies.  *Please list the excluded articles and the reasons for exclusion of each article in a supplementary file.* | Methods - subjects |
| 5 | Provide details on how the quality of the studies included in the analyses was assessed. | All data were collected and analyzed by the 6 individual studies (File S1 and ref 15), QC of genotyping in methods - genotyping and quality control |
| 6 | Describe steps taken to contact study authors to identify additional studies and to request missing data. | NA - all studies were part of the GABRIEL consortium |
| 7 | Describe how environmental effects were adjusted for, if this adjustment was not conducted, outline the reasons for this. | Methods - statistical analyses |
| 8 | Describe the methods of handling heterogeneity/between-study variance. | Methods - statistical analyses |
| 9 | Describe how the Hardy-Weinberg equilibrium and linkage disequilibrium were assessed. | HWE: Methods - genotyping and quality control. LD: NA |
| 10 | Describe and justify the choice of model for the analyses (per-allele vs per-genotype vs genetic model-free, random effects vs fixed effects). | Methods - statistical analyses |
| 11 | Describe whether a sensitivity analysis has been completed. | NA |
| 12 | Describe whether an assessment of the effects of population stratification has been conducted. | NA - only adjustment for informative principal components for within-Europe diversity (methods - statistical analyses) |
| 13 | Describe whether study-specific results have been assessed and if so the reasons for this (e.g. forest plot). | Methods - statistical analyses. Forest plots in results - figure 1, 2, and 3 |
|  | **Results** |  |
| 14 | Include flow diagram for the studies included in the meta-analysis as the first figure for the manuscript | NA - of the 23 studies in Gabriel we included 6 based on the availability of data on adult onset asthma. No other selection was applied. It is not necessary to show this in a figure |
| 15 | Report details on allele/genotype prevalence. | Results - table 2, 3, and 4 |
| 16 | Report the effect size estimates and p values for each analysis. | Results - table 2, 3, 4, and 5 |
|  | **Discussion** |  |
| 17 | Discuss the limitations of the meta-analysis, including genotyping errors/bias and publication bias. | NA - all studies were selected before the study-specific analyses and the meat-analyses were performed. All 6 studies were included in the meta-analysis. |
| 18 | If the meta-analysis identifies an association within a subgroup of the population studied but not another, discuss the implications of these results, and if applicable the possibility of subgroup-specific publication bias. | Results - table 5. Publication bias: NA |
| 19 | Discuss the suitability of the sample size employed to the research question and the power of the study. | Discussion - 4th and 7th paragraph |
